# Circ-0000197 derived from porcine milk small extracellular vesicles promotes intestinal barrier function by sponging miR-429

**DOI:** 10.1186/s40104-025-01218-5

**Published:** 2025-06-25

**Authors:** Yuxuan Wang, Bilan Chen, Tingzhou Xuan, Kun Ouyang, Jingshen Chen, Hailong Wang, Junyi Luo, Jiajie Sun, Qianyun Xi, Yongliang Zhang, Ting Chen

**Affiliations:** College of Animal Science, Guangdong Province Key Laboratory of Animal Nutritional Regulation, National Engineering Research Center for Breeding Swine Industry, State Key Laboratory of Livestock and Poultry Breeding, South China Agricultural University, Guangzhou, 510642 Guangdong China

**Keywords:** CircRNA, Intestinal barrier, Milk small extracellular vesicles, MiRNA, Occludin, ZO-1

## Abstract

**Background:**

As an essential source of nutrients for young mammals, milk possesses a variety of biological functions. Recently identified milk-derived small extracellular vesicles (sEV) have shown potential regulatory effects on intestinal health. Current studies have highlighted the functional roles of milk-derived sEV and their RNA cargo in promoting intestinal health. However, there is a paucity of research demonstrating how milk-derived sEV influence intestinal barrier function through the transport of circRNAs.

**Results:**

In this study, we aimed to investigate the effects of porcine milk sEV (PM-sEV) circRNA on intestinal barrier function. We systematically identified the circRNAs involved in this process and analyzed the miRNAs through which PM-sEV deliver circRNAs to regulate intestinal barrier function. Our findings revealed that PM-sEV promote the expression of the intestinal tight junction proteins ZO-1 and Occludin, both in vivo (mice) and in vitro (IPEC-J2). When PM-sEV RNA was reduced using ultrasound treatment, their ability to enhance intestinal barrier function was significantly reduced. Bioinformatics analysis showed that circ-0000197, present in PM-sEV, can target miR-429, while miR-429 has the ability to target the 3'-UTR of ZO-1 and Occludin. Furthermore, experiments involving the overexpression or inhibition of the relevant non-coding RNAs (ncRNAs) demonstrated that circ-0000197 significantly enhances intestinal barrier function, whereas miR-429 exerts an inhibitory effect on this function. Overall, our findings identify circ-0000197 in PM-sEV as a crucial circRNA that regulates intestinal barrier function by inhibiting miR-429. Circ-0000197 carried by PM-sEV acts as a competing endogenous RNA (ceRNA) that regulates the expression of ZO-1 and Occludin by sponging miR-429, thereby promoting intestinal barrier function at both the cellular and in vivo levels.

**Conclusions:**

These findings emphasize the vital role of circRNAs transported through milk-derived sEV in regulating intestinal health, offering new avenues for developing innovative functional milk components. This mechanism also underscores the importance of PM-sEV carrying circ-0000197 in preserving intestinal barrier integrity. Collectively, this study enhances our understanding of the complex regulatory networks involving PM-sEV carrying circRNAs and their impact on intestinal health.

**Supplementary Information:**

The online version contains supplementary material available at 10.1186/s40104-025-01218-5.

## Background

The gut serves as the primary site for digestion and absorption, as well as the largest immune organ in the body. The health and disease resistance of animals are closely associated with the function of the intestinal barrier [1, 2]. This barrier primarily comprised of small intestinal epithelial cells and the intercellular tight junction complex [3]. Tight junctions, located on the apical side of the intestinal epithelium, serve as the main connection points between these cells [4]. They play a crucial role in maintaining the polarity of intestinal epithelial cells and regulating the permeability of the intestinal mucosal barrier [5]. Tight junctions are composed of various proteins, including Occludin (OCLN), Claudins, and Zonula occludens (ZO), among others [6]. Notably, ZO-1 and OCLN are key components of tight junctions and are essential for strengthening tight junction barrier integrity [7].

Milk-derived small extracellular vesicles (sEV) are emerging as significant active components in milk, playing crucial roles in regulating intestinal health. These sEV are capable of transporting non-coding RNAs (ncRNAs), including miRNAs [8], lncRNAs [9], and circRNAs [10], from donor to recipient cells, thereby exerting various biological functions. Milk sEV have been shown to promote the growth of intestinal epithelial cells [11], alleviate intestinal inflammation [12], and protect against oxidative stress [13]. For instance, milk sEV carrying miR-146a-5p have been reported to promote early-life intestinal development in offspring [14]. In a model of dextran sulfate sodium (DSS)-induced intestinal epithelial cell injury, milk EV carrying miR-375 and miR-320 demonstrated the ability to reduce intestinal inflammation [15]. Similarly, in a model of lipopolysaccharide (LPS)-induced intestinal epithelial cell injury, miR-30a-5p carried by milk sEV was shown to mitigate inflammation in intestinal epithelial cells by targeting the NF-κB pathway [16, 17]. Similar to these findings, our previous research demonstrated that porcine milk sEV (PM-sEV) can improve intestinal barrier function in mice, with its miRNA alleviating LPS- and deoxynivalenol (DON)-induced damage to intestinal epithelial cells [18, 19]. In addition to promoting intestinal growth and alleviating intestinal inflammation, miR-34a carried by milk sEV has been shown to regulate the proliferation of intestinal cells under hypoxic conditions [20]. The delivery of miRNAs by milk-derived sEV plays a pivotal role in regulating intestinal health. Moreover, we found that circ-XPO4 carried by PM-sEV enhances the production of intestinal SIgA in piglets [21]. However, the role of circRNAs in PM-sEV in regulating intestinal barrier function remains unclear.

Up until now, numerous ncRNAs have been identified as playing significant roles in regulating the intestinal barrier, with miRNAs and lncRNAs comprising the majority of these molecules [22, 23]. Although the intestinal barrier has been extensively studied, our understanding of the regulatory function of circRNAs derived from PM-sEV in this context remains limited. Previous studies have indicated that one of the functions of circRNAs is to act as miRNA sponges, thereby counteracting miRNA-mediated mRNA degradation [24, 25]. Research has demonstrated that miR-429 impairs intestinal barrier function in mice by downregulating the expression of ZO-1 and OCLN proteins [26–28]. However, circRNAs derived from milk-derived sEV have not yet been shown to affect intestinal barrier function via miR-429. In this study, building on our previous sequencing analysis of circRNAs in PM-sEV, we predicted circRNAs that could be targeted by miR-429 and subsequently verified these predictions functionally. We further assessed whether circ-0000197 could regulate intestinal barrier function via the circ-0000197/miR-429/ZO-1 and OCLN axis, providing new insights into identifying functional components in PM-sEV that regulate intestinal health and opening new directions for the development of functional dairy products.

## Materials and methods

### Cells and plasmids

HEK 293T cells were purchased from the China Center for Type Culture Collection (CCTCC, Beijing, China). Professor Xiuqi Wang of South China Agricultural University generously provided the IPEC-J2. Circ-0000197 small interfering RNAs (siRNAs), miR-429 mimics, and inhibitor were designed and synthesized by GenePharma (Shanghai, China). The pCD2.1-ciR-circ-0000197 overexpression plasmid was synthesized by Guangzhou IGE Biotechnology Co., Ltd. (Guangzhou, China).

### EV extraction and EV RNA removal

Fresh porcine milk was obtained from healthy Landrace within 3–5 d after delivery at a pig farm in Guangdong, China. PM-sEV were isolated according to our previously reported method [21, 29–31]. The sEV concentration was quantified and expressed as mg total protein/mL. Subsequently, EV RNA was removed with reference to reported methods [32]. Briefly, PM-sEV were ultrasonicated for 1.5 h and then incubated for 1 h at 37 °C. RNA was then extracted, and the removal of circ-0000197 and miR-429 was identified by PCR and agarose gel electrophoresis.

### Identification of PM-sEV

The morphology of milk sEV was observed by transmission electron microscopy (TEM; JEM2000EX, JEOL, Tokyo, Japan). Briefly, the milk sEV sample of the appropriate concentration was set at the copper grid coated with formvar for 2 min, negatively stained with 1% uranyl acetate, observed, and photographed with TEM. The size of sEV was analyzed by nanoparticle tracking analysis (NTA; LM10-HS, Nanosight, Amesbury, UK). Milk sEV preparations were examined as described previously [33] with constant flow injection. Three recordings of 60 s each were captured. The biomarkers of sEV were detected by Western blot (described as follows). The representative sEV markers, CD9 (AP68965, Abcepta, China) and Alix (D262028, Sangon Biotech, Shanghai, China) were detected.

### Animal experiments and sample collection

The animal study was reviewed and approved by the Institutional Animal Care and Use Committee of South China Agricultural University, China. All animal experimentation complied with the laboratory animal management and welfare regulations approved by the Standing Committee of Guangdong People’s Congress (Guangzhou), China. Ethical code number: SCAU-AEC-2010-0416. For mouse experiments, 40 weaned Kunming male mice (21 d of age) were purchased from Guangdong Medical Laboratory Animal Center (Guangzhou, China). The mice were randomly allocated into 5 groups, with 8 mice in each group, which were control group (gavaged 200 μL PBS per mouse), the 1 mg sEV group, 1 mg sEV after ultrasound, 3 mg sEV group, and 3 mg sEV after ultrasound, the volume of gavage in each group was 200 μL. Each mouse was kept separately in a cage, housed in a room with a temperature of 25 ± 2 °C, a photoperiod of 12/12 h (day/night), and a relative humidity of 60% ± 10%. All mice had free access to water and food. Over the following 21 d, PM-sEV (containing 1 mg or 3 mg protein) were administered orally by gavage to mice, while the control group mice were administered PBS of the same volume each day. On d 22, the mice were euthanized, and their intestinal tissues were collected for further analysis. These samples were frozen in liquid nitrogen and stored at −80 °C for quantitative real-time PCR (qRT-PCR) and Western blotting analyses.

### RNA isolation and real-time PCR analysis

The method used for the RNA extraction has been described previously [34]. Briefly, total RNA was isolated by TRIzol reagent (Invitrogen, CA, USA) according to the manufacturer’s protocol. The cDNAs were obtained by Color Reverse Transcription Kit (EZBioscience, CA, USA). Quantification of mRNA, miRNA and circRNA was performed by a SBRY Green PCR Kit (Takara, Shiga, Japan). The circRNA and mRNA levels of cell and tissue samples were normalized to β-actin, sEV circRNA levels were normalized to cel-miR-39-3p, and the miR-429 levels were normalized to the *U6* and determined by 2^−△△Ct^ method. The primer sequences used in this study were presented in Table 1.

**Table 1:** Primer sequences for PCR

Gene	Forward primer (5´→3´)	Reverse primer (5´→3´)
mmu-Claudin-1	CATCAATGCCAGGTATGAATT	TGTTGGGTAAGAGGTTGTTT
mmu-*ZO-1*	CCTCCTGAGTTTGATAGTGGC	TCTCTCGGCAGACCTTGAA
mmu/ssc-*Ocln*	GCACCCAGCAACGACATA	CACATCACGATAACGAGCATA
mmu/ssc-*U6*	CTCGCTTCGGCAGCACA	AACGCTTCACGAATTTGCGT
D-CIRC-197	GAAACCTGAAGGGAAGCCAGA	TCGCTGGAAATCCATGATGTGAA
C-CIRC-197	AGCTGGTGGAACCATTTGGA	GTCTGGCTTCCCTTCAGGTT
ssc-*ZO-1*	TCACACCAAAACCATACAC	CGCCACTATCAAACTCAG
ssc-Claudin-1	TCAATACAGGAGGGAAGCCAT	ATATTTAAGGACCGCCCTCTCC
ssc-miR-429	AGTGCAGGGTCCGAGGTATT	CGCGCGTAATACTGTCTGGTAA
mmu/ssc-actin	TGCTGTCCCTGTATGCCTCT	CTTTGATGTCACGCACGATTT
cel-miR-39-3p	GGGTCACCGGGTGTAAATC	CAGTGCGTGTCGTGGAGT
ssc-miR-429 RT	GTCGTATCCAGTGCAGGGTCCGAGGTATTCGC	
cel-miR-39-RT	GTCGTATCCAGTGCGTGTCGTGGAGTCGGCAATTGCACTGGATACGACCAAGCT	

### miRNA mimics/miRNA inhibitor/plasmid transfection and RNA interference

The sequences of miRNA mimics/inhibitor and siRNA are as follows: 5´-UAAUACUGUCUGGUAAUGCCGU-3´ for miR-429 mimics, 5´-ACGGCAUUACCAGACAGUAUUA-3´ for miR-429 inhibitor, 5´-GGAUGAAAAGUCAGGAAACTT-3´ for siRNA-1, 5´-AAGUCAGGAAACUAGCCGATT-3´ for siRNA-2, 5´-GUCAGGAAACUAGCCGAGUTT-3´ for siRNA-3, and the control was the scrambled NC sequence provided by GenePharma (Shanghai, China). Mimics, siRNA and plasmid were transformed using the Lipofectamine 2000 (Invitrogen, CA, USA) according to the manufacturer’s instructions. Briefly, 1 × 10^5^ cells/mL IPEC-J2 were plated on a 12-well-plate and grown to about 70% to 80% confluence, using Lipofectamine 2000 transfection. For each well, 40 pmoL mimics/siRNA or 1.5 μg DNA were each diluted with 50 μL of FBS and PS free DMEM, 2 μL Lipofectamine 2000 were diluted with 50 μL of FBS and PS free DMEM, and incubated for 20 min in a mixture. IPEC-J2 were replenished with 900 μL DMEM, and the transfection mixture was added to each well dropwise. After 6 h transfection, the medium was replaced with a fresh medium (10% FBS and 1% PS) and continually cultured for a further 48 h.

### Western blot analysis

The method used for Western blot assay has been described previously [35]. Briefly, 1 mmol/L of PMSF (Thermo Fisher, MA, USA) was added to the RIPA lysis buffer, and the mixture was added to the cell samples. The samples were centrifuged at 12,000 × *g *and 4 °C for 10 min, and the supernatants were used to quantitative protein by the Pierce BCA Protein Assay Kit (Thermo Fisher, MA, USA). Denatured protein was electrophoresed in sodium dodecyl sulfate-polyacrylamide gel electrophoresis gels and transferred to a PVDF membrane. After incubation in a blocking buffer (5% nonfat milk powder in TBST) for 1 h at room temperature, the membrane was reacted with primary specific antibodies overnight at 4 °C and HRP-conjugated secondary antibodies for 1 h at room temperature. ImageJ software (National Institutes of Health, MD, USA) was used for gray scan analysis. The antibody against ZO-1 (21773-1-AP) was purchased from Proteintech (Wuhan, China). The antibody against OCLN (ab31721) was purchased from abcam (Cambridge, UK). The antibody against Claudin-1 (A21971) and GAPDH were purchased from ABclonal (Wuhan, China).

### Dual-Luciferase reporter assay

The target gene prediction was conducted through the starBase, lntaRNA, and TargetScan databases [36–38]. Further targeted verification was performed by dual-luciferase reporter assay, and HEK 293T cells were seeded in 96-well cell culture plates. When their confluence reached about 80%, the miR-429 mimic and pmirGLO-circ-0000197-WT/Mut (circ-0000197-WT/Mut) (Youkang, Guangzhou, China) were co-transfected into cells using Lipofectamine 2000. After incubation for 48 h, the cells were washed with PBS and the luciferase activity was measured by the Dual-GLO luciferase reporter assay system (Promega, WI, USA) to determine whether there was a target relationship between miR-429 and circ-0000197. The circ-0000197-WT/Mut sequences were presented in Table S1.

### Statistical analysis

The normality and homogeneity of variance were confirmed using Shapiro-Wilk test and Levene's test, respectively. Statistics was performed using Student’s *t*-test for two group and one-way analysis of variance (ANOVA) followed by LSD post hoc test using SPSS 21.0 (SPSS Inc., Chicago, IL, USA). And all data were expressed as means ± SEM.

## Results

### Identification of PM-sEV

**Fig. 1:**
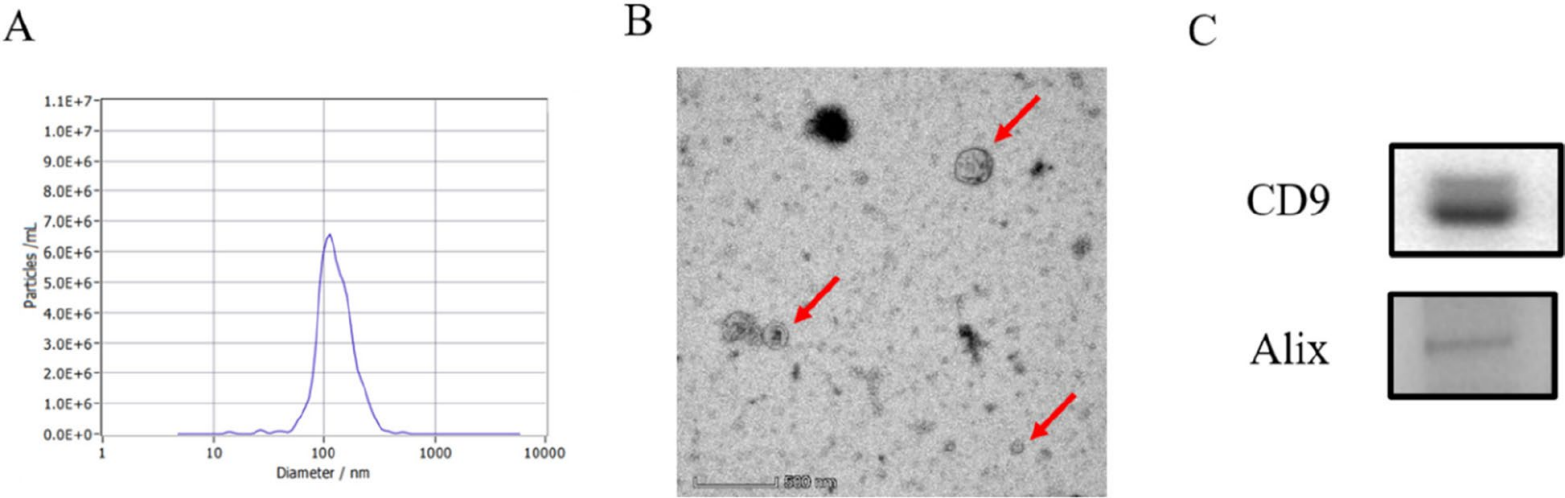
Identification of PM-sEV. **A** NTA of the isolated PM-sEV. **B** TEM image of the isolated PM-sEV. **C** Marker proteins of PM-sEV were detected by Western blot

We identified the isolated sEV by TEM, NTA, and marker protein detection via Western blot. TEM images revealed that PM-sEV had cup-like morphology (Fig. 1A). The distribution curve of the particle size of PM-sEV was determined by NTA (Fig. 1B), and the mean nanoparticle size of isolated PM-sEV is 135.1 nm. In addition, representative sEV markers CD9 and Alix were found in PM-sEV (Fig. 1C). These results show that we successfully isolated PM-sEV.

### Effects of PM-sEV on the viability and barrier function of IPEC-J2

**Fig. 2:**
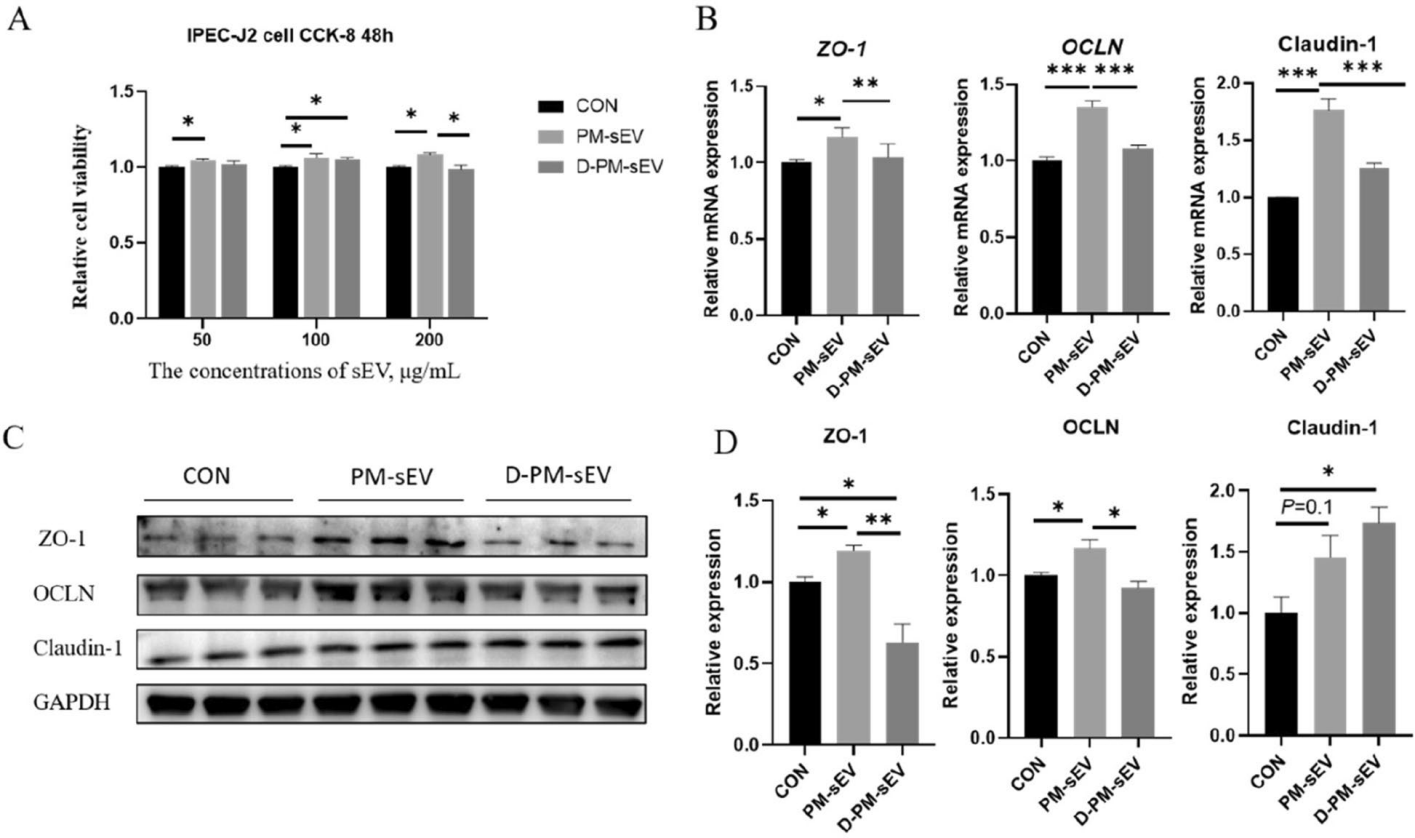
Effects of PM-sEV on the viability and barrier function of IPEC-J2. **A** CCK8 kit was used to screen the concentration of PM-sEV used on IPEC-J2 (*n* = 8). **B** The mRNA expression levels of *ZO-1*, *OCLN*, and Claudin-1 in IPEC-J2 (*n* = 4). **C** and **D** The protein expression levels of ZO-1, OCLN, and Claudin-1 in IPEC-J2 cells (*n* = 3). ^*^P < 0.05; ^**^P < 0.01; ^***^P < 0.001

To explore the effects of PM-sEV and its functional components on the viability of IPEC-J2, we treated IPEC-J2 with various concentrations of PM-sEV and PM-sEV after RNA removal by ultrasound (D-PM-sEV). The results demonstrated that ultrasonic treatment effectively removed most of the RNA content from PM-sEV (Fig. S1A). Notably, 200 μg/mL of PM-sEV significantly enhanced IPEC-J2 viability, whereas 200 μg/mL of D-PM-sEV showed no appreciable effect on cell viability (Fig. 2A). Consequently, we selected a concentration of 200 μg/mL of PM-sEV to further assessed its impact on IPEC-J2. Analysis of intestinal barrier related genes revealed that PM-sEV significantly increased the mRNA expression levels of *ZO-1*, *OCLN*, and Claudin-1, while D-PM-sEV did not produce a significant effect (Fig. 2B). Moreover, the protein expression levels of ZO-1 and OCLN were significantly upregulated following treatment with PM-sEV (Fig. 2C and D). Collectively, these results suggest that PM-sEV not only enhances the viability of IPEC-J2 but also promotes the expression levels of tight junction proteins. Based on the results, we speculate that the RNA components contained within PM-sEV appear to play a critical role in mediating these biological effects.

### Identification of PM-sEV circ-0000197 and verification of the target gene of circ-0000197

**Fig. 3:**
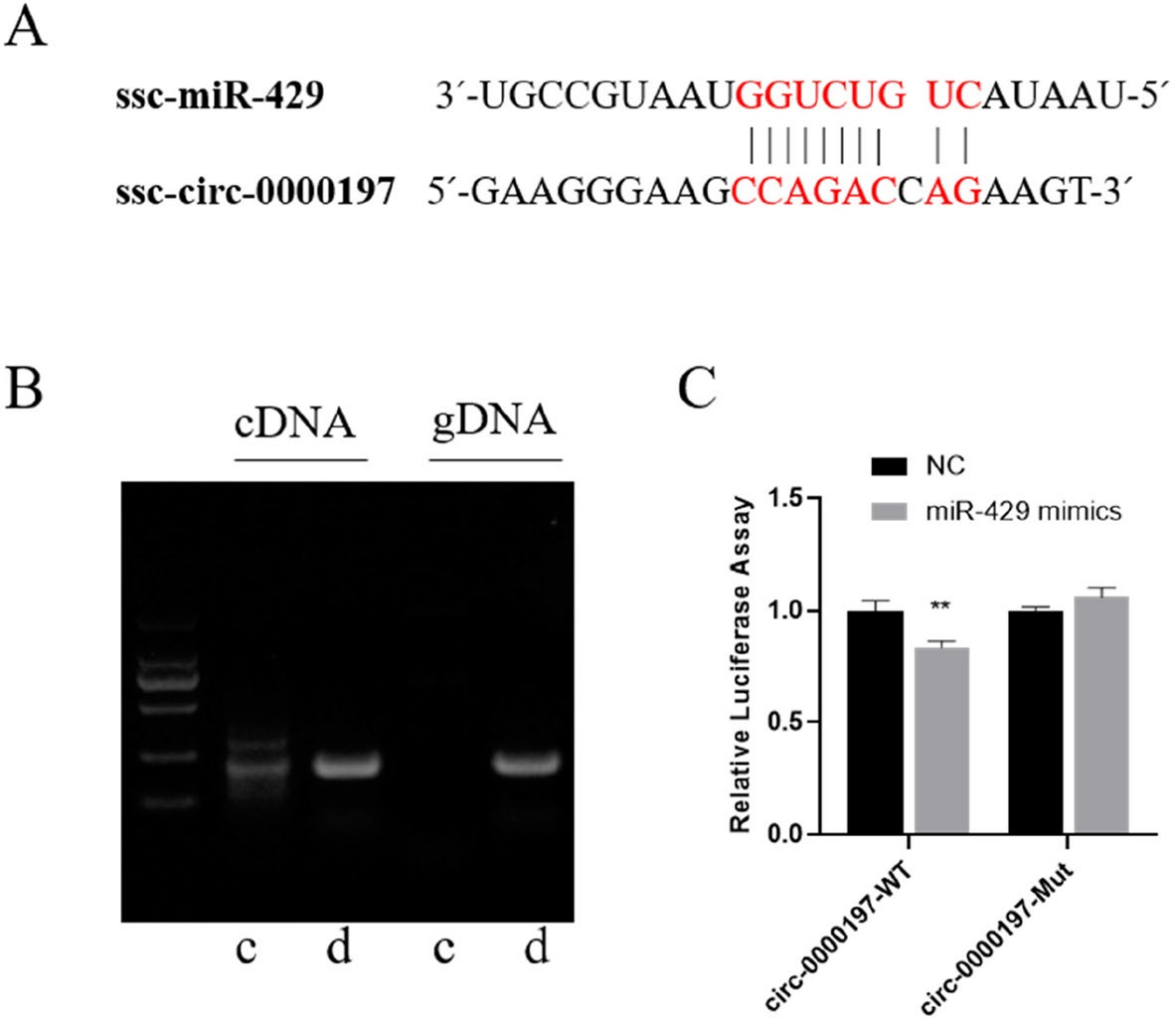
Verification of circRNA-miRNA-mRNA axis, and circ-0000197 was identified. **A** Putative binding sites of miR-429 to circ-0000197. **B** circ-0000197 was amplified from PM-sEV cDNA and gDNA using convergent and divergent primers. **C** Dual luciferase reporter gene experiment to verify the relationship between miR-429 and circ-0000197 (*n* = 8). ^*^P < 0.05; ^**^, *P* < 0.01; ^***^P < 0.001

Since of our sequencing results showed that exist circRNA in the PM-sEV [39], and the previously report have shown that miR-429 can target *ZO-1* and *OCLN* [26–28]. To further investigate this, we conducted a bioinformatics analysis and found that circ-0000197 within PM-sEV has the potential to regulate *ZO-1* and *OCLN* by binding to miR-429 (Fig. 3A). Since milk sEV originate mainly from the mammary gland [40], to confirm the presence of circ-0000197, we found that the convergent primer successfully amplified circ-0000197 from both cDNA and gDNA extracted from porcine mammary gland tissue, whereas the divergent primer can only amplified circ-0000197 from cDNA but not from gDNA (Fig. 3B). Sanger sequencing further validated the head-to-tail splicing characteristic of circ-0000197 in both porcine mammary gland tissue and PM-sEV (Fig. S1B). And circ-0000197 was shown to be resistant to RNase digestion (Fig. S1C), confirming its stability and presence in PM-sEV. To investigate the relationship between circ-0000197 and miR-429, we conducted a dual-luciferase reporter assay in HEK 293T cells, and the results showed that overexpression of miR-429 significantly downregulated luciferase activity in the circ-0000197-WT group compared to the control group (NC). Furthermore, the luciferase activity returned to levels consistent with the NC group when the binding site of circ-0000197 was mutated (circ-0000197-Mut) (Fig. 3C), indicating that circ-0000197 can indeed target miR-429. Those findings suggest that circ-0000197 is present in PM-sEV and can target miR-429, while miR-429 could target *ZO-1* and *OCLN*. This regulatory pathway highlights the potential biological significance of circ-0000197 in the context of PM-sEV and its role in modulating intestinal barrier functions.

### Overexpression of circ-0000197 promoted the expression of intestinal tight junction proteins

**Fig. 4:**
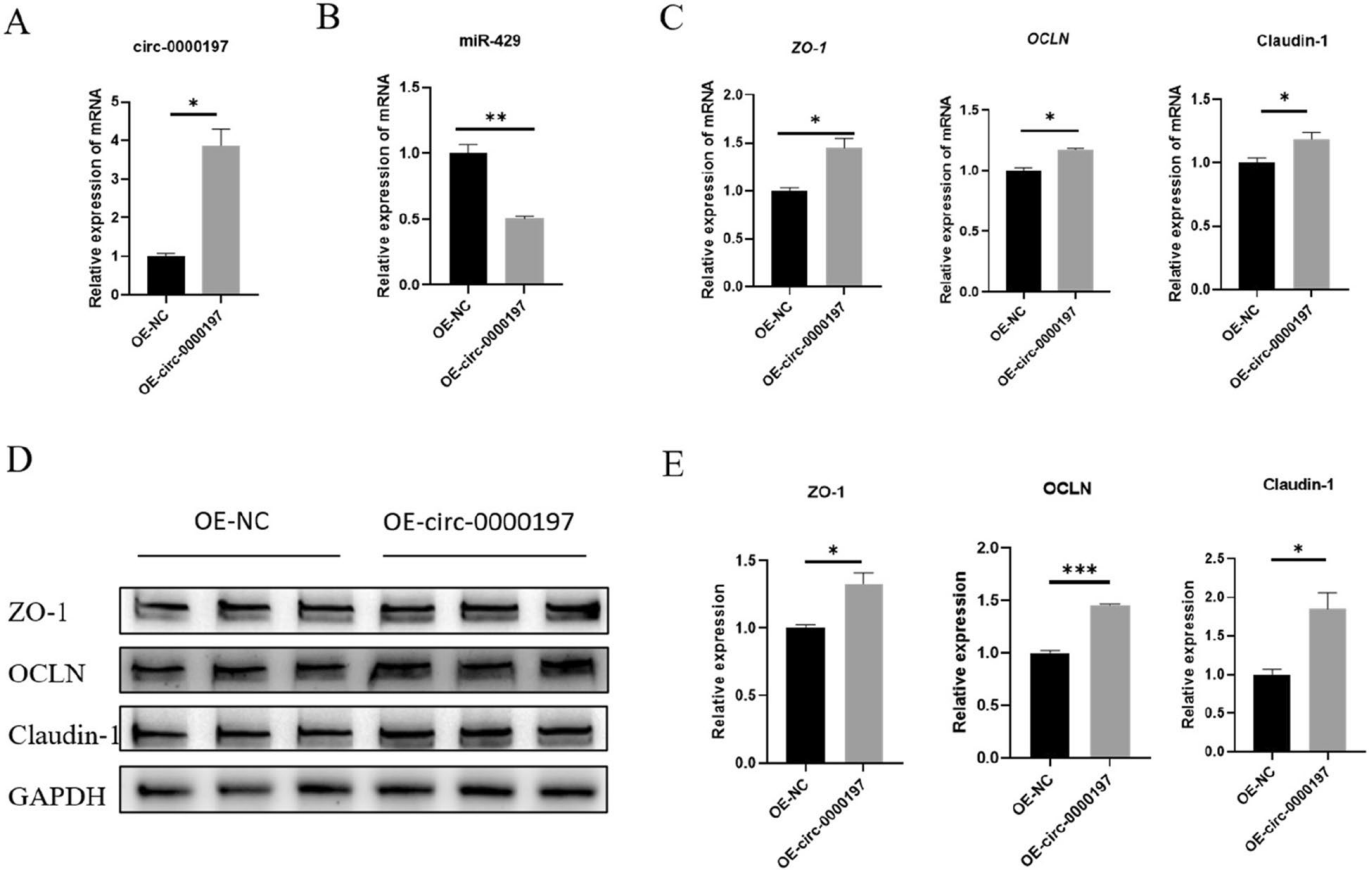
Overexpression of circ-0000197 could inhibit miR-429, promote the tight junction protein expression. **A** Expression level of circ-0000197 after overexpression of circ-0000197 (*n* = 3). **B** Expression level of miR-429 after overexpression of circ-0000197 (*n* = 3). **C** The mRNA expression levels of *ZO-1*, *OCLN*, and Claudin-1 after overexpression of circ-0000197 (*n* = 3). **D** and **E** The protein expression levels of ZO-1, OCLN, and Claudin-1 after overexpression of circ-0000197 (*n* = 3). ^*^P < 0.05; ^**^, *P* < 0.01; ^***^P < 0.001

To investigate whether circ-0000197 in PM-sEV could promote intestinal barrier function, we transfected an overexpression vector OE-circ-0000197 into IPEC-J2. Following transfection, we measured the mRNA expression levels of intestinal barrier related genes. The results indicated that the expression of circ-0000197 was significantly increased in the transfected cells (Fig. 4A), while the expression level of miR-429 was significantly decreased (Fig. 4B). Following circ-0000197 overexpression, TEER was significantly increased (Fig. S1D). Furthermore, the mRNA expression levels of the target genes *ZO-1* and *OCLN* were significantly increased (Fig. 4C). And the mRNA expression level of Claudin-1 also showed a significantly increased (Fig. 4C). Correspondingly, the protein expression levels of ZO-1, OCLN, and Claudin-1 reflected these observed mRNA changes (Fig. 4D and E). Collectively, these results hint that circ-0000197 can upregulate the intestinal tight junction proteins function via miR-429, highlighting the crucial role of circ-0000197 in regulating intestinal barrier integrity.

### Interfering with circ-0000197 inhibited the expression of intestinal tight junction proteins

**Fig. 5:**
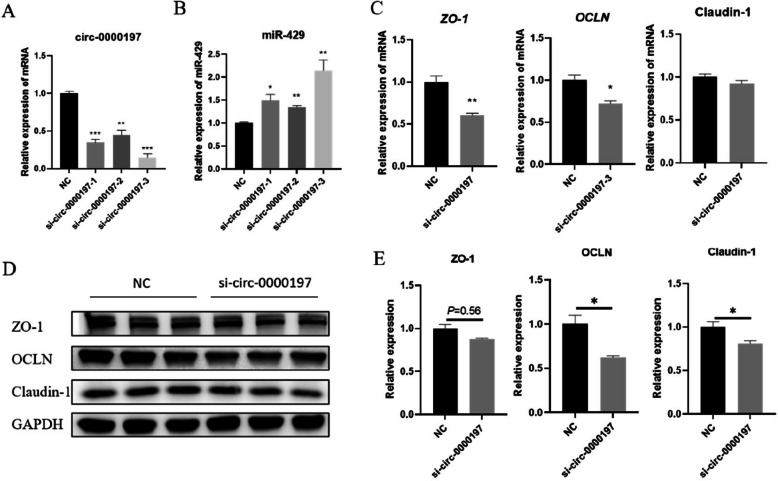
Interfering with circ-0000197 inhibited the expression of tight junction protein in IPEC-J2. **A** Circ-0000197 siRNA interference efficiency (*n* = 3). **B** Expression level of miR-429 after treated with si-circ-0000197 (*n* = 3). **C** The mRNA expression levels of *ZO-1*, *OCLN*, and Claudin-1 after treated with si-circ-0000197 (*n* = 3). **D** and **E** The protein expression levels of ZO-1, OCLN, and Claudin-1 after treated with si-circ-0000197 (*n* = 3). ^*^P < 0.05; ^**^P < 0.01; ^***^P < 0.001

To further explore the role of circ-0000197 in regulating IPEC-J2 barrier function, we designed and synthesized three siRNAs. Among these, si-circ-0000197-3 demonstrated superior efficiency in downregulating the expression of circ-0000197 compared to si-circ-0000197-1 and si-circ-0000197-2 (Fig. 5A), indicating that si-circ-0000197-3 was suitable for subsequent inhibition experiments. Following the silencing of circ-0000197 with si-circ-0000197, we observed a significant increase in the expression level of miR-429 (Fig. 5B). Additionally, circ-0000197 inhibition resulted in a significant decrease in TEER (Fig. S1E). Correspondingly, the mRNA expression levels of its target genes *ZO-1* and *OCLN* were significantly decreased (Fig. 5C). Moreover, the mRNA expression level of Claudin-1 showed a trend toward downregulation, although it did not reach statistical significance (Fig. 5C). The protein expression levels of OCLN and Claudin-1 were significantly decreased, while ZO-1 exhibited a trend toward downregulation (Fig. 5D and E). Overall, these results indicated that the inhibition of circ-0000197 leads to an upregulation of miR-429 and a subsequent decrease in the expression of intestinal tight junction protein ZO-1 and OCLN. Furthermore, our data suggest that circ-0000197 may also influence the expression of Claudin-1 through additional regulatory pathways. These findings underscore the role of circ-0000197 in modulating intestinal barrier function via its interaction with miR-429.

### Effect of miR-429 on intestinal tight junction proteins

**Fig. 6:**
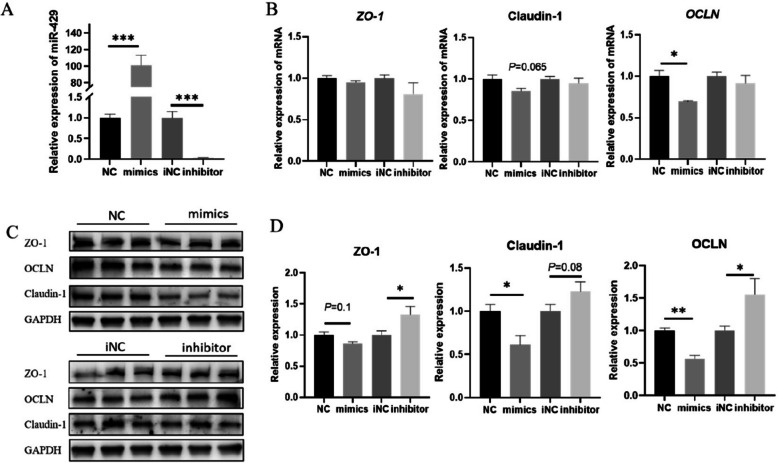
Effect of miR-429 on intestinal tight junction proteins. **A** miR-429 overexpression and inhibition efficiency (*n* = 3). **B** The mRNA expression levels of *ZO-1*, *OCLN*, and Claudin-1 after overexpression and inhibition of miR-429 (*n* = 3). **C** and **D** Protein expression levels of ZO-1, OCLN, and Claudin-1 after overexpression and inhibition of miR-429 (*n* = 3). ^*^P < 0.05; ^**^P < 0.01; ^***^P < 0.001

Given that circ-0000197 can promote intestinal barrier function and target miR-429, we investigated the regulatory effect of miR-429 on the expression levels of intestinal tight junction proteins. We first overexpressed and inhibited miR-429 in IPEC-J2 (Fig. 6A). The results showed that TEER was significantly reduced following miR-429 overexpression (Fig. S1F), accompanied by a significant reduction in the mRNA and protein expression level of OCLN (Fig. 6B–D). Conversely, inhibition of miR-429 led to a significant increase in TEER (Fig. S1G), as well as a significant rise in the mRNA and protein expression levels of OCLN (Fig. 6B–D). In addition to its effects on OCLN, the protein level of Claudin-1 was significantly decreased, while the protein level of ZO-1 exhibited a trend towards downregulation, although this was not statistically significant after miR-429 overexpression (Fig. 6C and D). Meanwhile, following the inhibition of miR-429, the protein level of ZO-1 was significantly increased, and the protein level of Claudin-1 showed an upward trend, albeit without statistical significance (Fig. 6C and D). Interestingly, despite these changes in protein expression, no significant differences were observed in the mRNA expression levels of *ZO-1* and Claudin-1 following miR-429 overexpression or inhibition (Fig. 6B). Furthermore, the prediction results of starBase and Targetscan database showed that there was no binding target between miR-429 and Claudin-1 (Table S2 and S3). These results collectively suggest that miR-429 can directly target and inhibit the protein expression of ZO-1 and OCLN, while also downregulating Claudin-1 through non-target-dependent pathways. Overall, those results indicate a complex regulatory role for miR-429 in modulating the expression of intestinal tight junction proteins, highlights its potential involvement in regulating intestinal barrier integrity.

### Effects of PM-sEV circ-0000197 on growth and intestinal tract of mice

**Fig. 7:**
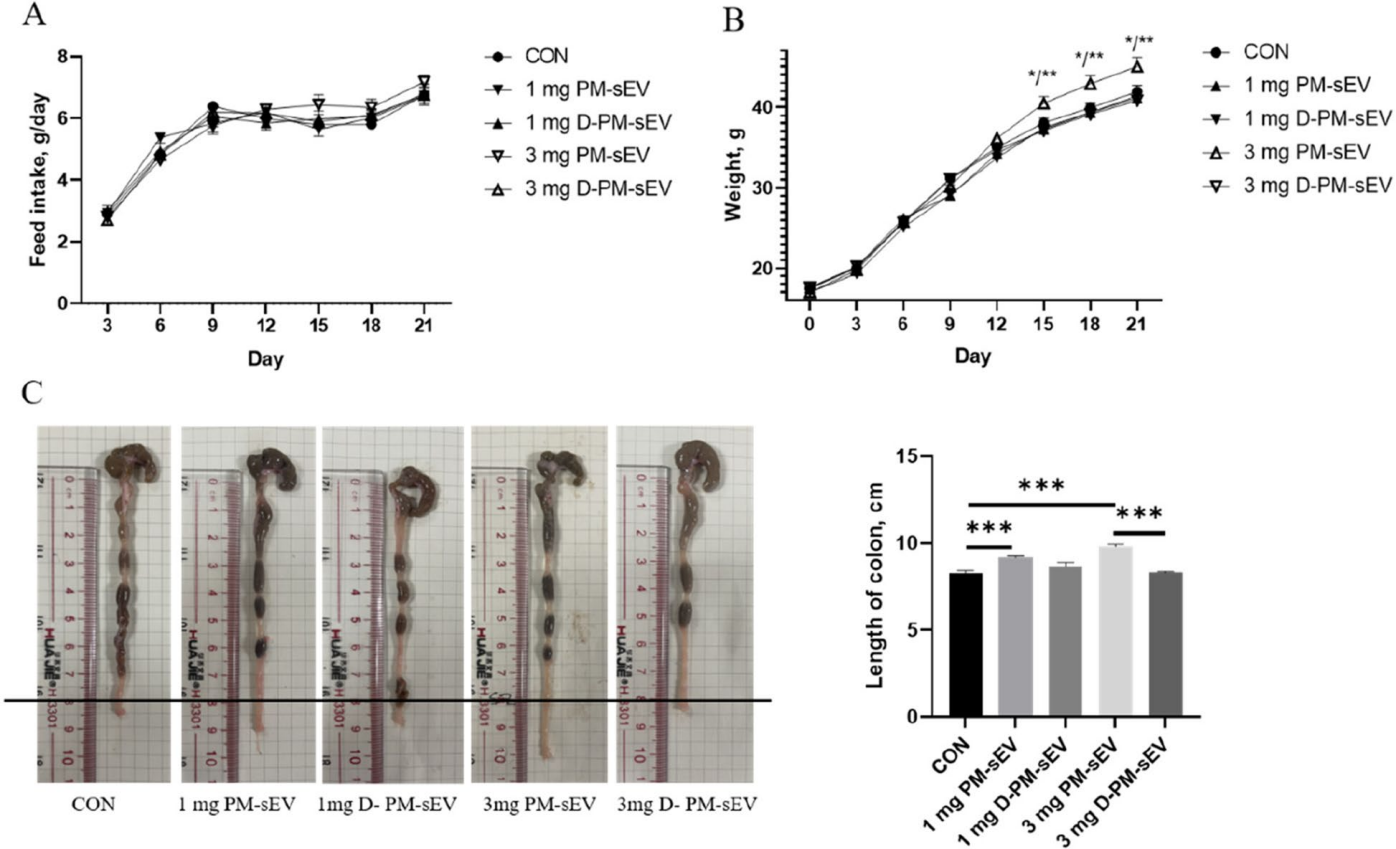
Effects of PM-sEV circ-0000197 on growth and intestinal tract of mice. **A** Feed intake of mice (*n* = 8). **B** Body weight of mice (*n* = 8). **C** Length of colon in mice (*n* = 8). ^*^P < 0.05; ^**^P < 0.01; ^***^P < 0.001

To further validated the effect of PM-sEV circ-0000197 in vivo, we administered PM-sEV and D-PM-sEV to the mice via gavage. The results displayed no significant difference in food intake among the groups (Fig. 7A). However, notable differences were observed in body weight, specifically, the body weight of the 3 mg PM-sEV group was significantly higher than that of the control group and the 3 mg D-PM-sEV group on d 15, 18, and 21 (Fig. 7B). This finding suggests that PM-sEV may enhance nutrients uptake or conversion in the mice. Besides, PM-sEV positively influenced intestinal development, as evidenced by the increased colon length. For instance, we observed longer colon lengths in the 1 mg and 3 mg PM-sEV groups compared to the control group, with the 3 mg PM-sEV showing the most pronounced effect (*P* < 0.001, Fig. 7C). In addition, the colon length in the 3 mg PM-sEV group was significantly greater than that in the 3 mg D-PM-sEV group (Fig. 7C). Collectively, these findings indicate that PM-sEV not only enhances body weight and nutrient absorption but also promotes intestinal development through improved morphological characteristics of the colon.

### Effect of PM-sEV circ-0000197 on intestinal tight junction proteins in mice

**Fig. 8:**
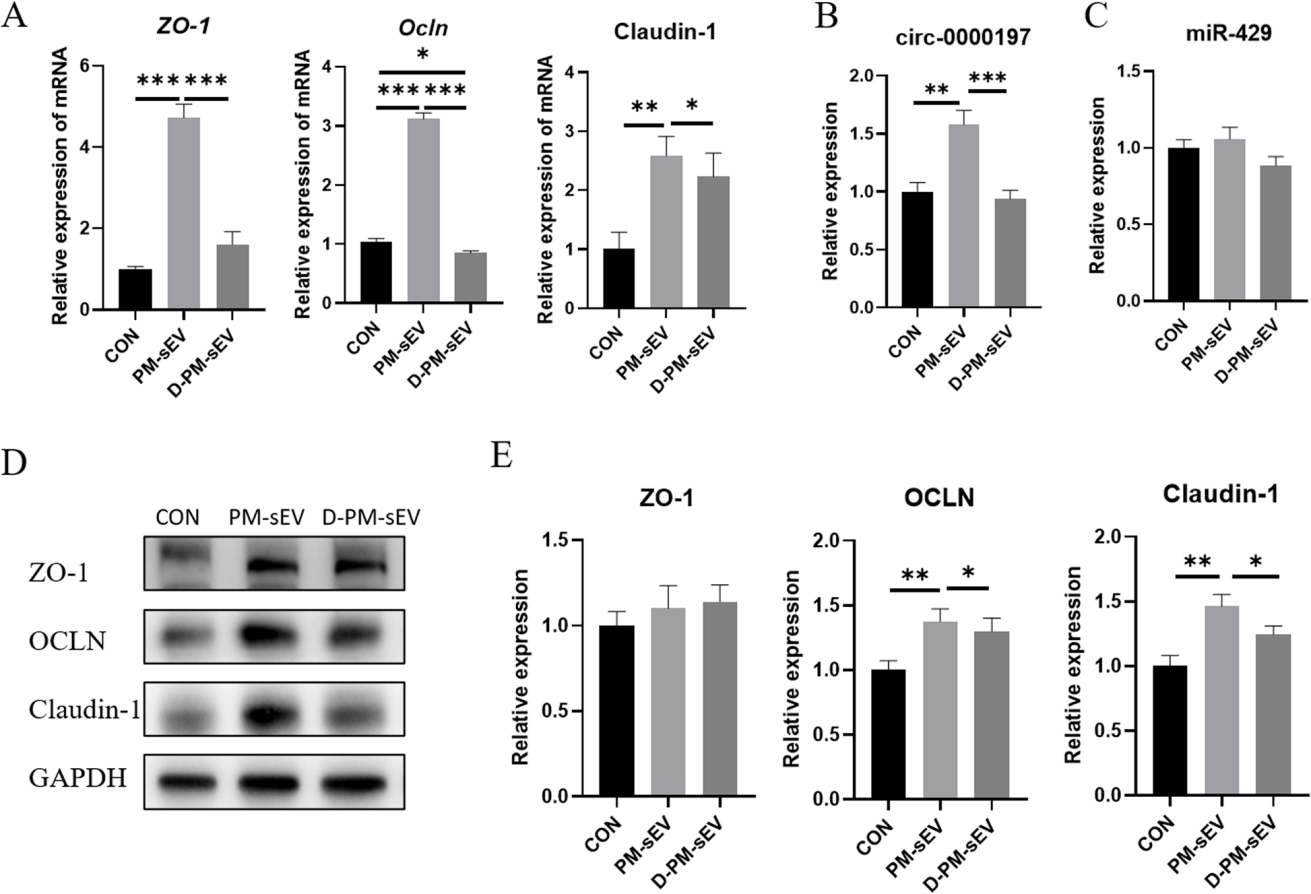
Effect of PM-sEV circ-0000197 on intestinal tight junction proteins in mice. **A** The mRNA expression levels of *ZO-1*, *Ocln*, and Claudin-1 after gavage of PM-sEV (*n* = 8). **B** Expression level of circ-0000197 in the jejunum of mice (*n* = 8). **C** Expression level of miR-429 in the jejunum of mice (*n* = 8). **D** and **E** The protein expression levels of ZO-1, OCLN, and Claudin-1 after gavage of PM-sEV (*n* = 8). ^*^P < 0.05; ^**^P < 0.01; ^***^P < 0.001

To further identified the regulatory effect of PM-sEV circ-0000197 on intestinal tight junction proteins, we examined the expression levels of circ-00000197 and tight junction proteins in the jejunum of mice in the 3 mg PM-sEV and D-PM-sEV groups. The results showed that the ultrasonic treatment decreased circ-0000197 expression in PM-sEV (Fig. S1A). Concurrently, circ-0000197 levels were significantly higher in the intestines of mice from the PM-sEV group compared to those in the control and D-PM-sEV groups (Fig. 8B). Regarding the expression of circ-0000197 targets and intestinal barrier-related molecules, the mRNA expression levels of *ZO-1*, *OCLN*, and Claudin-1 in the 3 mg PM-sEV group were significantly higher than those in the control group (Fig. 8A), Additionally, the protein expression levels of OCLN and Claudin-1 in the 3 mg PM-sEV group were significantly increased, whereas the protein level of ZO-1 did not show a significant increase (Fig. 8D and E). Notably, the expression level of the target miR-429 exhibited no significant difference across all groups (Fig. 8C). These data indicate that PM-sEV can regulate intestinal barrier function by delivering circ-0000197 in vivo, and that circ-0000197 may regulate the expression of barrier-related proteins through mechanisms beyond the circ-0000197/miR-429 axis. This suggests a more complex regulatory mechanism governing intestinal tight junction integrity mediated by PM-sEV.

## Discussion

The intestinal barrier serves as the first line of defense against foreign substances in the gastrointestinal tract [41]. The functionality of the intestinal mucosal barrier primarily relies on the integrity of tight junction proteins within the intestinal epithelial cells. A reduction in the levels of these proteins can increase intestinal permeability, ultimately leading to dysfunction of the intestinal mucosal barrier [42]. Key tight junction proteins, such as OCLN, Claudin-1, and ZO-1, are essential structural components that tightly regulate the permeability of molecules across the barrier [43, 44]. Studies have shown that enhancing the expression levels of OCLN, Claudin-1, and ZO-1 can help preserve the integrity of the intestinal barrier [45–47]. Previous research has indicated that milk-derived sEV can influence intestinal barrier function. For instance, human breast milk-derived sEV been found to protect epithelial tight junction proteins, including ZO-1, Claudin, and OCLN, from inflammatory damage [48]. Furthermore, miR-148 derived from bovine and human milk sEV has been shown to mitigate intestinal epithelial cell injury by upregulating OCLN and ZO-1 [49]. In this study, we found that PM-sEV enhance the viability of IPEC-J2 and promote the expression of intestinal tight junction proteins both in vivo (mice) and in vitro (IPEC-J2) (Figs. 2 and 8), thereby improving intestinal barrier function. However, despite these promising findings, the specific molecules within PM-sEV that play a key regulatory role in this process remain unidentified.

The components of milk-derived sEV are complex and include proteins, nucleic acids, lipids, and other constituents. Among these, ncRNAs are important components of milk-derived sEV [50–53]. While much of the research concerning milk sEV ncRNAs has focused on miRNA [54, 55], studies on milk sEV circRNAs remain limited. The main mode of action of circRNA is to serve as sponges for miRNAs, thereby targeting and eliminating the inhibitory effects of miRNAs on their target genes [56–58]. Some studies have demonstrated that miR-429 can regulate intestinal barrier function by targeting ZO-1 and OCLN [26–28]. Furthermore, in addition to its targeted inhibition of ZO-1 and OCLN, overexpression of miR-429 has been shown to suppress the expression of barrier-related protein Claudin-5 [59]. which were similar results obtained in the present study. In this study, we found that circ-0000197 present in PM-sEV specifically targets miR-429. Notably, the overexpression of circ-0000197 led to the inhibition of miR-429, which in turn promoted the protein expression of OCLN, ZO-1, and Claudin-1 in IPEC-J2 (Fig. 4). In contrast, overexpression of miR-429 resulted in the inhibition of protein expression for both OCLN and Claudin-1 (Fig. 6). Further validation through dual luciferase assays confirmed that circ-0000197 directly targets miR-429 (Fig. 3). Based on these observations, we propose that circ-0000197 present in PM-sEV can regulate intestinal barrier function through the circ-0000197/miR-429/ZO-1 and OCLN axes. This pathway highlights the potential of circRNAs in modulating intestinal health.

In addition to functioning through the circRNA/miRNA/mRNA axis, circRNAs can also exert their biological functions through alternative pathways. Notably, some circRNAs may possess coding potential, which allows them to influence biological processes directly [60, 61]. Furthermore, miRNAs can regulate gene expression not only by directly targeting mRNAs but also by interacting with other functional proteins to enhance protein expression levels, thereby exerting distinct biological functions [62]. miR-328 has been found to act as a decoy by binding to regulatory RNA-binding proteins and preventing them from inhibiting mRNA translation. Thus, miR-328 serves a dual role in the regulation of gene expression [63]. In this study, we found that circ-0000197 could also regulate the expression levels of tight junction protein Claudin-1 in IPEC-J2 (Figs. 4 and 5). Interestingly, Claudin-1 was not predicted to target miR-429 according to analyses using the starBase and Targetscan databases (Table S2 and S3), however, our results demonstrated that overexpression of circ-0000197 led to increased levels of Claudin-1 protein in IPEC-J2 (Fig. 4). Conversely, overexpression of miR-429 inhibited the Claudin-1 protein within the same cellular context (Fig. 6). Additionally, in vivo, although we observed a significant increase in circ-0000197 expression, there was no significant difference in miR-429 levels (Fig. 8). In the circRNA/miRNA/mRNA axis, circRNAs act as sponges that can sequester miRNAs from their mRNA targets, implying buffering activity rather than degradation [64]. This leads us to speculate that circ-0000197 may exert its biological function by competitively inhibiting miRNA binding to target genes in complex in vivo assays. It is also possible that circ-0000197 affect the intestinal barrier function through pathways other than circ-0000197/miR-429/ZO-1 and OCLN axes, which remain to be elucidated. Moreover, it is possible that various functional substances present in PM-sEV contribute to this dynamic regulatory process. Overall, our findings suggest a complex interplay between circRNA, miRNA, and protein expression, warranting further investigation to elucidate the underlying mechanisms. This led us to speculate that circ-0000197 may exert its biological function by competitively inhibiting miRNA binding to target genes in complex in vivo assays, which may be circ-0000197 competitive inhibition of miRNA binding to target genes to exert biological functions. It is also possible that circ-0000197 affects intestinal barrier function through pathways other than circ-0000197/miR-429/ZO-1 and OCLN axes, which remains to be elucidated. In addition, various functional substances present in PM-sEV may contribute to this dynamic regulatory process. In summary, our findings suggest a complex interplay between circRNA, microRNA and protein expression, and further studies are needed to elucidate the underlying mechanisms.

## Conclusions

In summary, this study reveals the molecular mechanism by which circ-0000197 in PM-sEV regulates intestinal barrier function through the circ-0000197/miR-429/ZO-1 and OCLN axes for the first time. These findings provide new insights into the exploration of novel functional components of milk-derived sEV and their regulatory mechanisms related to intestinal health. Furthermore, these insights may pave the way for the development of therapeutic strategies aimed at enhancing intestinal barrier function through the modulation of circRNA and sEV components, as well as exploring the potential applications of these findings in clinical settings.

## Supplementary Information

Supplementary Material 1: Table S1 The sequences of circ-0000197-WT/Mut and Ocln-WT/Mut.

Supplementary Material 2: Table S2 starBase database.

Supplementary Material 3: Table S3 TargetScan database.

Supplementary Material 4: Fig. S1A circ-0000197 content in PM-sEV after ultrasonic treatment. Fig. S1B Sanger sequencing further validated the head-to-tail splicing characteristic of circ-0000197 in both porcine mammary gland tissue and PM-sEV. Fig. S1C The amplified products were processed by Rnase. Fig. S1D TEER changes after overexpression of circ-0000197 (*n *= 3). Fig. S1E TEER changes after inhibition of circ-0000197 (*n *= 3). Fig. S1F TEER changes after overexpression of miR-429 (*n *= 3). Fig. S1G TEER changes after inhibition of miR-429 (*n *= 3).

## Data Availability

The datasets used and/or analyzed during the current study are available from the corresponding author on reasonable request.

## Abbreviations

ceRNA: Competing endogenous RNA
DON: Deoxynivalenol
D-PM-sEV: PM-sEV for removal of RNA by ultrasound
DSS: Dextran sulfate sodium
LPS: Lipopolysaccharide
ncRNAs: Non-coding RNAs
OCLN: Occludin
PM-sEV: Porcine milk small extracellular vesicles
sEV: Small extracellular vesicles
siRNA: Small interfering RNAs
ZO: Zonula occludens
