# Supplementary material for: Circ-0000197 derived from porcine milk small extracellular vesicles promotes intestinal barrier function by sponging miR-429
Source: J Anim Sci Biotechnol. 2025 Jun 25;16:89. doi: 10.1186/s40104-025-01218-5 (PMC12188651; doi:10.1186/s40104-025-01218-5)
Supplement: Supplementary file 1 — Supplementary Material 1: Table S1 The sequences of circ-0000197-WT/Mut and Ocln-WT/Mut. [file 40104_2025_1218_MOESM1_ESM.docx]

**Table S1** The sequences of circ-0000197-WT/Mut and Ocln-WT/Mut

circ-0000197-WT (5´ to 3´): GCTAGCCTTGAGATACCAACTATTACAGCTGGTGGAACCATTTGGAGTTATTTCAAATCATCTGATTCTAAATAAAATTAATGAGGCGTTCATTGAAATGGCAACCACAGAAGATGCTCAGGCTGCAGTGGATTATTATACAACCACACCAGCATTAGTATTTGGCAAGCCAGTGAGAGTTCATTTATCCCAGAAGTATAAAAGAATAAAGAAACCTGAAGGGAAGCCAGACCAGAAGTTTGATCAAAAGCAAGAGCTTGGACGTGTGATACATCTCAGCAATTTACCTCATTCTGGCTATTCTGACAGTGCTGTCCTCAAGCTTGCTGAGCCTTATGGAAAAATAAAGAATTATATACTAATGAGGATGAAAAGCTCGAG

circ-0000197-Mut (5´ to 3´): GCTAGCCTTGAGATACCAACTATTACAGCTGGTGGAACCATTTGGAGTTATTTCAAATCATCTGATTCTAAATAAAATTAATGAGGCGTTCATTGAAATGGCAACCACAGAAGATGCTCAGGCTGCAGTGGATTATTATACAACCACACCAGCATTAGTATTTGGCAAGCCAGTGAGAGTTCATTTATCCCAGAAGTATAAAAGAATAAAGAAACCTGAAGGGAAGAAGTGAAGTAAGTTTGATCAAAAGCAAGAGCTTGGACGTGTGATACATCTCAGCAATTTACCTCATTCTGGCTATTCTGACAGTGCTGTCCTCAAGCTTGCTGAGCCTTATGGAAAAATAAAGAATTATATACTAATGAGGATGAAAAGCTCGAG

Ocln-WT(5´ to 3´): GCTAGCGAGGCAGAGGCCACCTTGTTTGAGAGATTAAGAAGTATCTGATCTGACATCTCTGCAGTGTTGTCAGGAGTCAAAATGACACTGGACTCTGACCCAGGAGGCCAAACCTTTCTGGTCATTACGGAGATTTGGTAGCTTTAATATCATCAGTATTGAAGCATTTTATAAATAGCTTTTGATAATCAACTGGTCTGAACACTCCAAGGAAGGATTTTTGTTTGTCTATCTGTTTTGTTTGTTTTTTAGGGCTGCCCCCATGCCATATGGAAGTTCCCAGGCTAGGGGTCTAAACTGAGCTACAGCTGCCGGCCTACGCCACAGCCACAGCAATGCCAGATCCTTCAACCCTCGAG

Ocln-Mut (5´ to 3´): GCTAGCGAGGCAGAGGCCACCTTGTTTGAGAGATTAAGAAGTATCTGATCTGACATCTCTGCAGTGTTGTCAGGAGTCAAAATGACACTGGACTCTGACCCAGGAGGCCAAACCTTTCTGGTCATTACGGAGATTTGGTAGCTTTAATATCATTGACGCCGAAGCATTTTATAAATAGCTTTTGATAATCAACTGGTCTGAACACTCCAAGGAAGGATTTTTGTTTGTCTATCTGTTTTGTTTGTTTTTTAGGGCTGCCCCCATGCCATATGGAAGTTCCCAGGCTAGGGGTCTAAACTGAGCTACAGCTGCCGGCCTACGCCACAGCCACAGCAATGCCAGATCCTTCAACCCTCGAG
