# Supplementary material for: Circ-0000197 derived from porcine milk small extracellular vesicles promotes intestinal barrier function by sponging miR-429
Source: J Anim Sci Biotechnol. 2025 Jun 25;16:89. doi: 10.1186/s40104-025-01218-5 (PMC12188651; doi:10.1186/s40104-025-01218-5)
Supplement: Supplementary file 4 — Supplementary Material 4: Fig. S1A circ-0000197 content in PM-sEV after ultrasonic treatment. Fig. S1B Sanger sequencing further validated the head-to-tail splicing characteristic of circ-0000197 in both porcine mammary gland tissue and PM-sEV. Fig. S1C The amplified products were processed by Rnase. Fig. S1D TEER changes after overexpression of circ-0000197 (n = 3). Fig. S1E TEER changes after inhibition of circ-0000197 (n = 3). Fig. S1F TEER changes after overexpression of miR-429 (n = 3). Fig. S1G TEER changes after inhibition of miR-429 (n = 3). [file 40104_2025_1218_MOESM4_ESM.docx]

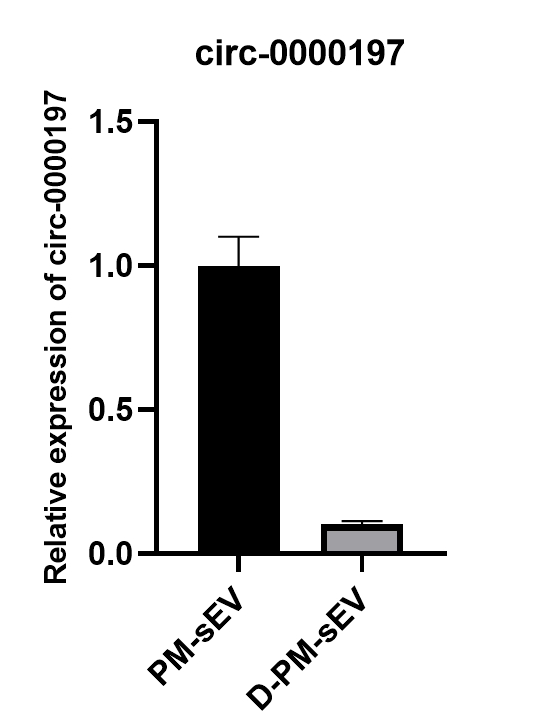


**Fig. S1A** circ-0000197 content in PM-sEV after ultrasonic treatment


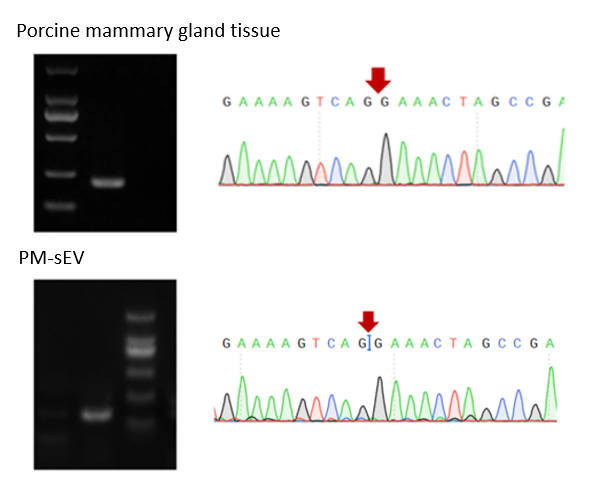


**Fig. S1B** Sanger sequencing further validated the head-to-tail splicing characteristic of circ-0000197 in both porcine mammary gland tissue and PM-sEV


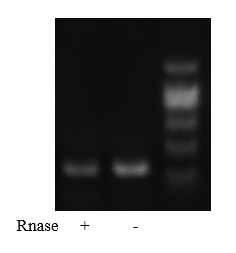


**Fig. S1C** The amplified products were processed by Rnase


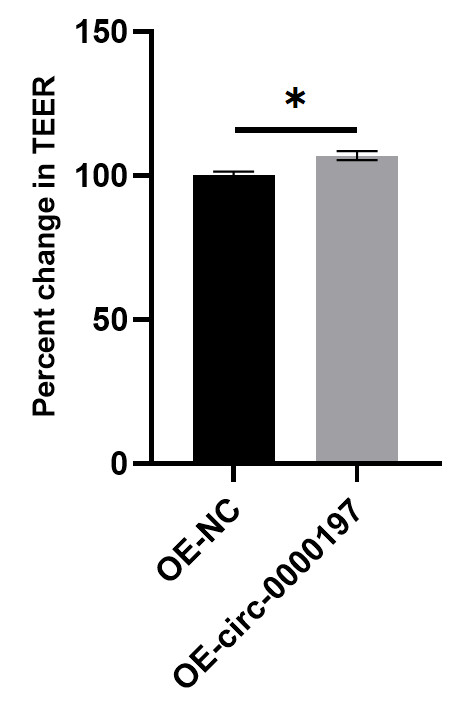


**Fig. S1D** TEER changes after overexpression of circ-0000197 (*n* = 3)


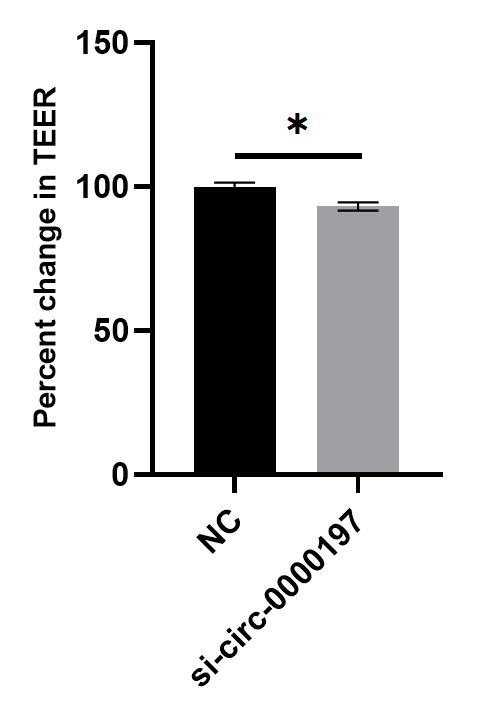


**Fig. S1E** TEER changes after inhibition of circ-0000197 (*n* = 3)


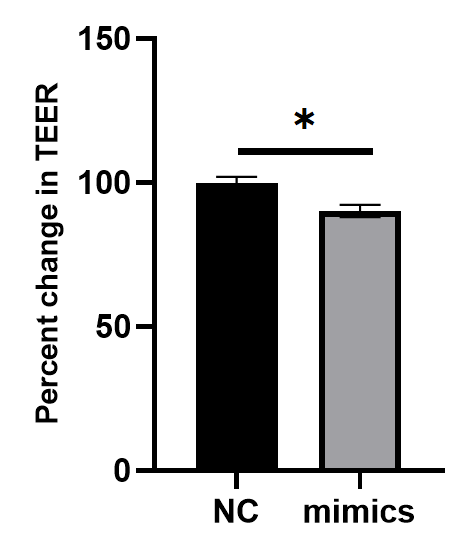


**Fig. S1F** TEER changes after overexpression of miR-429 (*n* = 3)


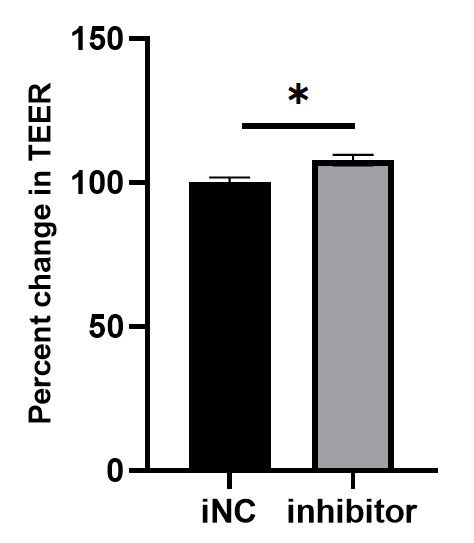


**Fig. S1G** TEER changes after inhibition of miR-429 (*n* = 3)
